# Scale‐dependent strategies for coexistence of mesocarnivores in human‐dominated landscapes

**DOI:** 10.1111/btp.12705

**Published:** 2019-09-22

**Authors:** David Carricondo‐Sanchez, Morten Odden, Abhijeet Kulkarni, Abi Tamim Vanak

**Affiliations:** ^1^ Faculty of Applied Ecology and Agricultural Sciences Inland Norway University Koppang Norway; ^2^ Ashoka Trust for Research in Ecology and the Environment Bangalore India; ^3^ School of Life Sciences University of KwaZulu‐Natal Durban South Africa; ^4^ DBT/Wellcome Trust India Alliance Program Hyderabad India

**Keywords:** anthropogenic disturbance, dog, habitat use, India, Indian fox, jackal, jungle cat, occupancy

## Abstract

Identifying factors influencing the distribution of and interactions within carnivore communities is important for understanding how they are affected by human activities. Species differ in their ability to adapt to humans depending on their degree of specialization in habitat use and feeding habits. This results in asymmetric changes in the ecology of co‐occurring species that can influence their interactions. We investigated whether human infrastructures and free‐ranging domestic dogs (a species typically associated with humans) influenced the co‐occurrence and habitat use of mesocarnivores in a landscape of high human population density in Maharashtra, India. We used 40 camera trap locations during 233 trapping nights and used Bayesian co‐occurrence occupancy models to investigate the habitat use and coexistence of species at different spatial scales. Additionally, we investigated their temporal overlap in space use. Indian foxes altered their habitat use both spatially and temporally in order to avoid free‐ranging domestic dogs and other larger competitors. The use of human infrastructure by jackals and jungle cats was limited by the presence of dogs. Our results illustrate how habitat use of smaller carnivore species changes both spatially and temporally in order to avoid larger competitors. We also show that the presence of species associated with humans mediates the influence of human infrastructures on the habitat use of mesocarnivores. We highlight the importance of acknowledging the potential impact of urbanization not only on single species, but also on the interactions within the community.

## 1 INTRODUCTION

Human‐dominated landscapes are expanding across the earth's surface. In these landscapes, the natural habitat is altered to give way to agricultural development and urban expansion (Saunders, Hobbs & Margules, 1991). Consequently, natural habitats turn into a fragmented mosaic of natural patches and cultivated land in which humans and wildlife coexist (Saunders et al., 1991). In order to minimize the impact of this expansion on biodiversity without compromising food production and development, two alternatives are debated: land sharing (i.e., biodiversity and human development sharing space) and land sparing (i.e., segregated spaces; Phalan, Onial, Balmford & Green, 2011; Tscharntke et al., 2012). However, determining which alternative is the most suitable requires a deep understanding of the different parts of the ecosystem. In this context, identifying potential changes in the behavior and habitat use of animals due to an altered natural habitat is particularly important in human‐dominated areas. This is especially true for mammalian carnivore species, whose exposure to historic and current human persecution may have increased their sensitivity to human disturbance (George & Crooks, 2006).

Changes in the natural habitat of a species and its exposure to human influence can affect its distribution, behavior, and habitat use. For example, cougars *Felis concolor* are negatively affected by habitat fragmentation and avoid paved roads (Beier, 1995; Dickson, Jenness & Beier, 2005). In North America, a number of species have experienced a reduction on their range due to a combination of human factors including population density, land use, and infrastructures (Laliberte & Ripple, 2004). Other species may benefit from human impact, for example, by an increased habitat carrying capacity through anthropogenic food subsidization. This is the case of the red fox *Vulpes vulpes* throughout its range (Bino et al., 2010; Carricondo‐Sanchez, Samelius, Odden & Willebrand, 2016; Elmhagen & Rushton, 2007), or raccoons *Procyon lotor* and coyotes *Canis latrans* in the USA (Ordenana et al., 2010; Prange, Gehrt & Wiggers, 2003). An increased food subsidization due to urban expansion can even benefit endangered species like the San Joaquin kit fox *Vulpes macrotis mutica,* which can inhabit areas of moderately dense human populations (Newsome, Ralls, Job, Fogel & Cypher, 2010). How much a species is affected by human influence will typically depend on the degree of specialization in its habitat use and feeding habits (Matthews, Cottee‐Jones & Whittaker, 2014).

Alterations of the natural habitat might also affect the intraguild interactions within the carnivore community (Linnell & Strand, 2000), both among native species (Berger & Gese, 2007; Lindstrom, Brainerd, Helldin & Overskaug, 1995; Palomares & Caro, 1999; Ritchie & Johnson, 2009) and with species commonly associated with humans, such as domestic cats *Felis catus* and dogs *Canis familiaris* (Vanak & Gompper, 2009). Changes in the intraguild interactions of a community may lead to spatial or temporal partitioning of the niche, segregating the habitat use of competitive species (Linnell & Strand, 2000; Schoener, 1974). Although some studies have focused on these interactions at a community level (Bu et al., 2016; Cruz, Sarmento & White, 2015; Farris, Kelly, Karpanty & Ratelolahy, 2016), there is limited information on how human alterations of the habitat might affect them (Gompper, Lesmeister, Ray, Malcolm & Kays, 2016; Lesmeister, Nielsen, Schauber & Hellgren, 2015; Rota et al., 2016). In particular, few studies have investigated the intraguild interactions in the mesocarnivore community in landscapes with very high human population density.

With over 1.3 billion inhabitants, India is the second‐most populous country in the world. It has a population density of 446 inhabitants per square kilometer (http://data.un.org/), and approximately 48% of the total land is dedicated to agriculture (http://www.fao.org/). In this context, native fauna have to coexist in a human‐altered landscape. Moreover, native fauna also have to contend with high densities of free‐ranging domestic dogs (Home, Bhatnagar & Vanak, 2018). Dogs in India can reach densities of 719 dogs/km^2^ in some areas (Belsare & Gompper, 2013), and they can affect native carnivores through interference competition, intraguild predation, and disease transmission (Vanak & Gompper, 2009). Despite the high human pressure on the environment, recent research has revealed a rich faunal diversity in areas of high human population density (Athreya, Odden, Linnell, Krishnaswamy & Karanth, 2013). The coexistence of humans with large carnivores and emblematic species is well documented in India (Athreya, Odden, Linnell, Krishnaswamy & Karanth, 2016; Chartier, Zimmermann & Ladle, 2011; Dhanwatey et al., 2013; Suryawanshi, Bhatia, Bhatnagar, Redpath & Mishra, 2014), but few ecological studies focus on the coexistence of mesopredators with humans at a community level.

In this study, we aimed to investigate whether human infrastructures and free‐ranging domestic dogs (a species typically associated with humans) influenced the co‐occurrence and habitat use of species in a mesocarnivore community in a landscape of high human population density in Maharashtra, India. We first used a multi‐species occupancy modeling in a Bayesian framework to investigate how habitat composition and human infrastructure affected the habitat use of Indian foxes *Vulpes bengalensis*, jungle cats *Felis chaus*, golden jackals *Canis aureus*, and domestic dogs, and how this habitat use varied across different spatial scales (Mayor, Schneider, Schaefer & Mahoney, 2009). Additionally, we used this framework to investigate whether dog activity had an impact on the probability of detecting a given species. Next, we investigated spatial and temporal niche partitioning among the four species. We predicted that dogs would be positively associated with humans and negatively associated with the other three species (Vanak & Gompper, 2009). We expected that jackals and jungle cats would be partially associated with human activities (Jaeger, Haque, Sultana & Bruggers, 2007; Nowell & Jackson, 1996), whereas Indian foxes would be more closely associated with natural habitats (Vanak & Gompper, 2010). Given the larger body mass of jungle cats, jackals, and dogs compared to that of the Indian fox, we predicted a negative association with Indian fox due to interference competition (Palomares & Caro, 1999).

## 2 METHODS

### 2.1 Study area

**Figure 1:**
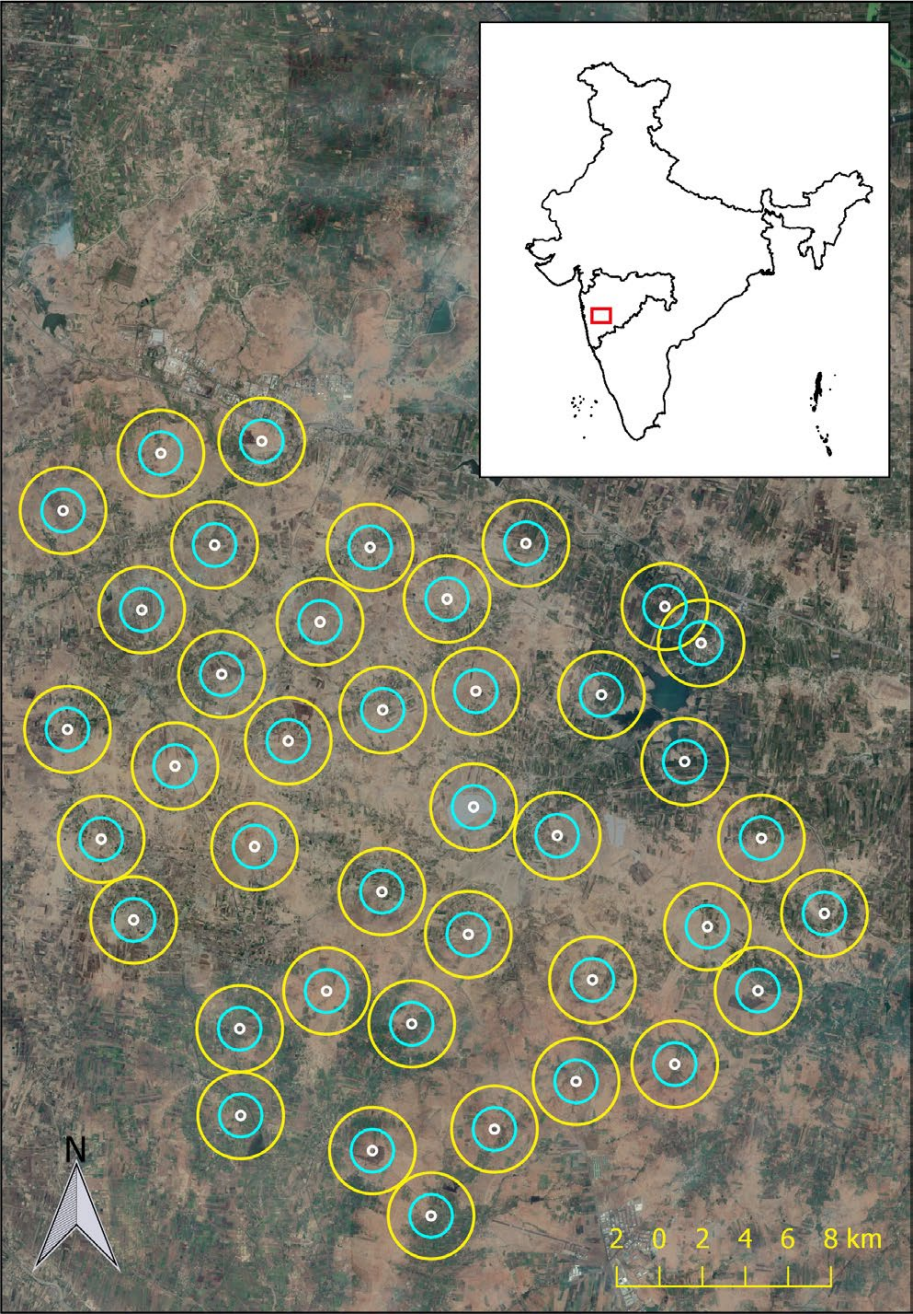
Study area located in the vicinity of Baramati, Maharashtra, India. Circles circumscribe the camera trap location and depict the buffer areas from which habitat data was extracted (white = 100 m buffer size, blue = 500 m buffer size, yellow = 1,000 m buffer size)

Our study area in Maharashtra, central India (Figure 1), covered ~230 square kilometers and included several villages that belong to the tehsils of Baramati and Daund, with population densities of 397/km^2^ and 380/km^2^, respectively (www.census2011.com). The elevation ranges from 546 to 643 m above mean sea level. The area has a semi‐arid climate with a cool‐dry season from November to February, a hot‐dry season from March to June, and a wet season from July to October during which 95% of the precipitation occurs.

The landscape consists of a matrix of sugarcane fields, seasonal crops, communal grazing lands, and forestry plantations. The latter typically consist of plantations of *Acacia* spp., *Azadirachta indica*,* Eucalyptus hybrid,* and *Glyricidia sepium*. Some remnant patches of grassland exist in the area, dominated by *Aristida* spp., *Heteropogon* spp., *Chrysopogon* spp., *Cymbopogon* spp., and *Dicanthium* spp. Natural scrub vegetation, like *Zizyphus mauritiana*,* Acacia leucophlea*,* and Acacia nilotica* is also present in the area. Common mesocarnivore species are Indian fox, golden jackal, and jungle cat. Palm civet *Paradoxurus hermaphrodites*, small Indian civet *Viverra zibetha*, grey mongoose *Herpestes edwardsii*, and herbivores like the Indian gazelle *Gazella bennettii* and Indian hare *Lepus nigricollis* are also common. Free‐ranging domestic dogs are found throughout the entire study area, either solitary or in packs.

### 2.2 Camera trapping

We recorded the activity of the study species during 6 weeks in the months of November and December of 2016 by deploying camera traps (ATC 28 trail cameras with IR led flash) at 40 trapping locations. We designed a systematic sampling regime using satellite imagery. To ensure that the whole area was covered, we first super‐imposed a grid of 2 km^2^ cell size on a map of the study area and applied a systematic sampling design by surveying alternative cells. Within each cell, we selected a potential suitable location that was accessible, minimized theft, and was spaced at least 2 km apart from the closest camera locations (except for two locations that were placed 1.2 km apart due to accessibility; Figure 1). Once in the field, we adjusted the locations within approximately 100 m from the original site to maximize the likelihood of detection of the target species (e.g., by using trails or natural paths) and minimize direct human disturbance. When adjusting the location, we ensured that the minimum distance between cameras was still at least 2 km. We noted when the cameras were placed on (*n *= 31) or off a trail (i.e., no trail or animal path within approximately 100 m around the camera location, *n *= 9). At each location, we attached and secured a camera trap to a tree at 30–50 cm height. We set up the cameras to take pictures every 5 s during periods of movement detection. At every trap location, we added some drops of lure (Red Fox Gland Lure; Carman's superior animal lures) between 1 and 2 m from the camera trap. We reapplied the lure every time the camera was deployed. The use of lures does not affect temporal activity, maximum movement distance, or immigration rates of species like leopard *Panthera pardus* or Malagasy civet *Fossa fossana* (Braczkowski et al., 2016; Gerber, Karpanty & Kelly, 2012). The carnivore community studied was naïve to the lure we used, and therefore, we assumed no a priori avoidance. Although the use of lures has not been tested in the study species, we did not observe any alteration of movement or temporal activity patterns of the study species (from ongoing GPS telemetry data; Vanak, A. T. unpublished).

GPS telemetry data from jungle cats and jackals in the area (A.T. Vanak, unpublished data) revealed that the average home ranges of these species exceed the separation between the cameras. Therefore, the study design violates the assumption of independence between camera locations. Accordingly, in this study, we investigated the habitat use rather than the occupancy of the study species.

The camera traps were active for six consecutive trapping nights. Preliminary studies in the area showed that, given the density of the target species in the area, four trapping nights were sufficient to ensure the detection of a species given presence in the area. In order to avoid theft, the camera traps were deployed between 16:30 and 18:30 hr every day and retrieved the next morning between 06:30 and 08:30 hr. Due to this constraint, we divided the study period in six blocks, managing six to seven cameras at a time in each block. The hours when the cameras were deployed accurately capture the maximum activity period of the study species (Boitani & Ciucci, 1995; Vanak & Gompper, 2007; A.T. Vanak, unpublished data). Moreover, human influence can affect the diel activity of wildlife by increasing their nocturnality (Gaynor, Hojnowski, Carter & Brashares, 2018). Therefore, we believe our data are a good representation of the habits and activity of these species.

### 2.3 Covariate data extraction

We retrieved satellite imagery of our study area from Google Earth (Google Earth, 2016), dated 15 November 2016, and digitized it by creating polygons or lines of the features of interest for our study (i.e., forestry plantation, agriculture land, fallow land, human settlements, water canals, and paved roads). In order to estimate characteristics of second‐, third‐, and fourth‐order habitat use (site, patch, and home range scales, respectively; after Johnson, 1980), buffers of 100 m radius, 500 m radius, and 1,000 m radius were created around each camera trapping location. Using zonal statistics tools, we extracted habitat data from the digitized map for each of the buffers around each camera location. We extracted the proportion of forest, agriculture, fallow land, and human settlements (buildings and population nucleus) in each buffer. As a proxy for habitat homogeneity, we calculated the average area of the habitat patches (i.e., forest, agriculture, fallow, or human settlement) within the buffer. Additionally, we calculated the accumulated length of paved roads and water canals within each buffer, and the distance from the camera to the closest human settlement patch. We used ArcGIS 10.3.1 (ESRI, 2009) for the digitization of satellite imagery and data extraction. We then standardized all continuous covariates by subtracting the mean and dividing by the standard deviation, and run Pearson's correlation test to check for collinearity. No pair of covariates showed high correlation values (i.e., >.60).

### 2.4 Spatial co‐occurrence

We created detection histories for Indian foxes, jackals, jungle cats, and domestic dogs. At each location *i,* we set a value of one (*y*_*sij*_ **= 1) if the species *s* was detected at least once during trapping night *j*, and zero (*y*_*sij*_ **= 0) if the species *s* was not detected during trapping night *j* (e.g., *y*_*si*_ **= 100,101; species *s* was detected in location *i* at least once during the first‐, fourth‐, and sixth‐trapping nights).

We used these histories to model habitat use and interactions among the studied species by fitting the multi‐species occupancy model described in Rota et al. (2016). MacKenzie et al. (2002) described a single‐species model to estimate the probability of a site being occupied when the detection probability of the species is less than one; the model from Rota et al. (2016) is a generalization of this single‐species model to include two or more interacting species. In contrast to other co‐occurrence models such as the one described in MacKenzie, Bailey and Nichols (2004), the interactions between species do not have to be asymmetrical. Moreover, the model allows the use of covariates to predict the probability that two or more species occupy the same site.

First, we modeled habitat use of the study species without including species interactions. For that, we started with a full occupancy model for each individual species (one for each buffer size) while keeping the rest of the species constant (i.e., intercept modeling). This step is, in essence, similar to creating one single‐species occupancy model for each species at each buffer size. The initial full model included habitat type and human infrastructure covariates. The best model for each species and buffer size was selected by following a backward selection approach by systematically retaining those effects where the 80% credible interval did not overlap zero (i.e., we were 90% certain of the direction of the effect). The final multi‐species model without species interactions was a combination of the best “single‐species” models (Tables S1–S3). We avoided placing the cameras close to roads to avert theft, and thus, we did not include the “Road” variable in the 100 m buffer size. Instead, we used the “Distance to settlement” variable. Given that “Distance to settlement” represents a value obtained from the specific location of the camera, we assumed that its effect was better revealed at the smallest buffer size. In order to model the detection probability of the four species, we used the “Trail” variable. Additionally, we included the number of dog occurrence events per trapping night at each site to investigate the potential influence of dog activity on the detection probability of fox, jackal, and jungle cat.

Next, we investigated the impact of human infrastructure variables (human settlements, length of canals, and length of tarmac roads/distance to settlements) on the co‐occurrence of the study species. We used the final multi‐species habitat use model for each buffer size to test whether the fit of the initial models improved by adding species interactions. The model selection approach was similar to the habitat use models (i.e., systematic backward selection for each pair of species).

We used non‐informative normal distributions with mean = zero, and standard deviation = 10 as priors in all the models. Models were fitted with the “rstan” package (Stan Development Team, 2018) in R 3.4.1 (R Core Team, 2017) by running three Monte Carlo Markov chains of 6,000 iterations each, discarding the first 2,000 as burn‐in process. We diagnosed the convergence of the models by visually checking trace plots and by using the Gelman and Rubin's converge diagnosis (Gelman & Rubin, 1992). We calculated WAIC values to evaluate model fit.

### 2.5 Activity patterns

We investigated the temporal interactions between the study species by creating and examining kernel density plots of the nocturnal activity (from ca. 17:00 to ca. 8:30) of each species and by calculating the coefficients of overlap of the different pairs of species (Ridout & Linkie, 2009).

To investigate the nocturnal activity of the species, we used the time of the events expressed as radians. Photographs of the same species taken within ten minutes were considered one single event. We created density curves of activity by calculating the kernel densities of the radian times and estimated the coefficients of overlap (∆) (Ridout & Linkie, 2009) by using the R package “Overlap” (Meredith & Ridout, 2017). The coefficient of overlap is an estimate of the overlap area in two density curves. Of five possible estimators of the coefficient of overlap, we chose the estimator ∆_1_ since it performs better for small sample sizes (*n* < 50) (Ridout & Linkie, 2009). We then used a bootstrapping technique to calculate the credible intervals of the overlapping coefficients (Ridout & Linkie, 2009). We used the coefficients of overlap and the visual inspection of the activity density plots to estimate the temporal interactions of the species.

## 3 RESULTS

### 3.1 Spatial co‐occurrence

During 233 trapping nights, we obtained 395 photographic events of the target species. Among the 40 locations, we recorded Indian foxes at least once in 14 locations (*n *= 52), jackals (*n *= 29) in eight, jungle cats (*n *= 34) in 15, and domestic dogs (*n *= 280) in 29 locations.

Dogs showed the highest occurrence (use) probabilities in our study area (0.725, 80% CRI [0.634, 0.813]), whereas jackals showed the lowest (0.246, 80% CRI [0.165, 0.331]). Indian foxes and jungle cats presented intermediate values (0.405, 80% CRI [0.321, 0.491] and 0.515, 80% CRI [0.373, 0.661], respectively).

All models performed better than the null model based on WAIC values (Table 1, Tables S1–S4). Moreover, WAIC values of the models with and without interactions were within two values apart for all the buffer sizes (Table 1).

**Table 1:** Structure and WAIC values of the best occupancy models explaining the habitat use and co‐occurrence of mesocarnivores in Maharashtra, India. *k* represents the number of parameters in each model. Δ WAIC represents the difference in WAIC between each model and the null model

	Model structure	*k*	WAIC	Δ WAIC
Model 1000 m	Fox: Intercept + Patch area + Settlements + Forest + Roads	10	708.345	18.920
	Jackal: Intercept + Patch area + Roads			
	Jungle cat: Intercept			
	Dog: Intercept			
w/ Interactions	Jackal & Dog: Intercept + Canals	14	710.553	16.712
	Jungle cat & Dog: Intercept + Roads			
Model 500 m	Fox: Intercept + Patch area + Settlements + Forest + Canals	12	690.783	36.482
	Jackal: Intercept + Patch area + Forest + Roads			
	Jungle cat: Intercept + Roads			
	Dog: Intercept			
w/ Interactions	Jackal & dog: Intercept + Canals	14	691.399	35.866
Model 100 m	Fox: Intercept	7	717.080	10.185
	Jackal: Intercept + Settlements			
	Jungle cat: Intercept + Settlements			
	Dog: Intercept + Settlements			
w/ Interactions	Indian fox & Jackal: Intercept	8	716.247	11.018
Null model	Fox: Intercept	10	727.265	0
	Jackal: Intercept			
	Jungle cat: Intercept			
	Dog: Intercept			

The probability of detection of the three wild species increased when the camera was placed on a trail (logistic coefficients for Indian fox: 0.532, 80% CRI [−0.604, 1.732]; jackal: 1.362, 80% CRI [0.446, 2.318]; Jungle cat: 1.536, 80% CRI [0.035, 2.949]), but it decreased for dogs (logistic coefficient: −0.788, 80% CRI [−1.282, −0.301]; Figure S1a). Dog activity had a negative effect on the detectability of foxes (logistic coefficient: −0.359, 80% CRI [−0.678, −0.052]), but not on the other species (logistic coefficients for jackal: 0.017, 80% CRI [−0.294, 0.330]; jungle cat, 0.174, 80% CRI [−0.059, 0.407]; Figure S1b).

**Figure 2:**
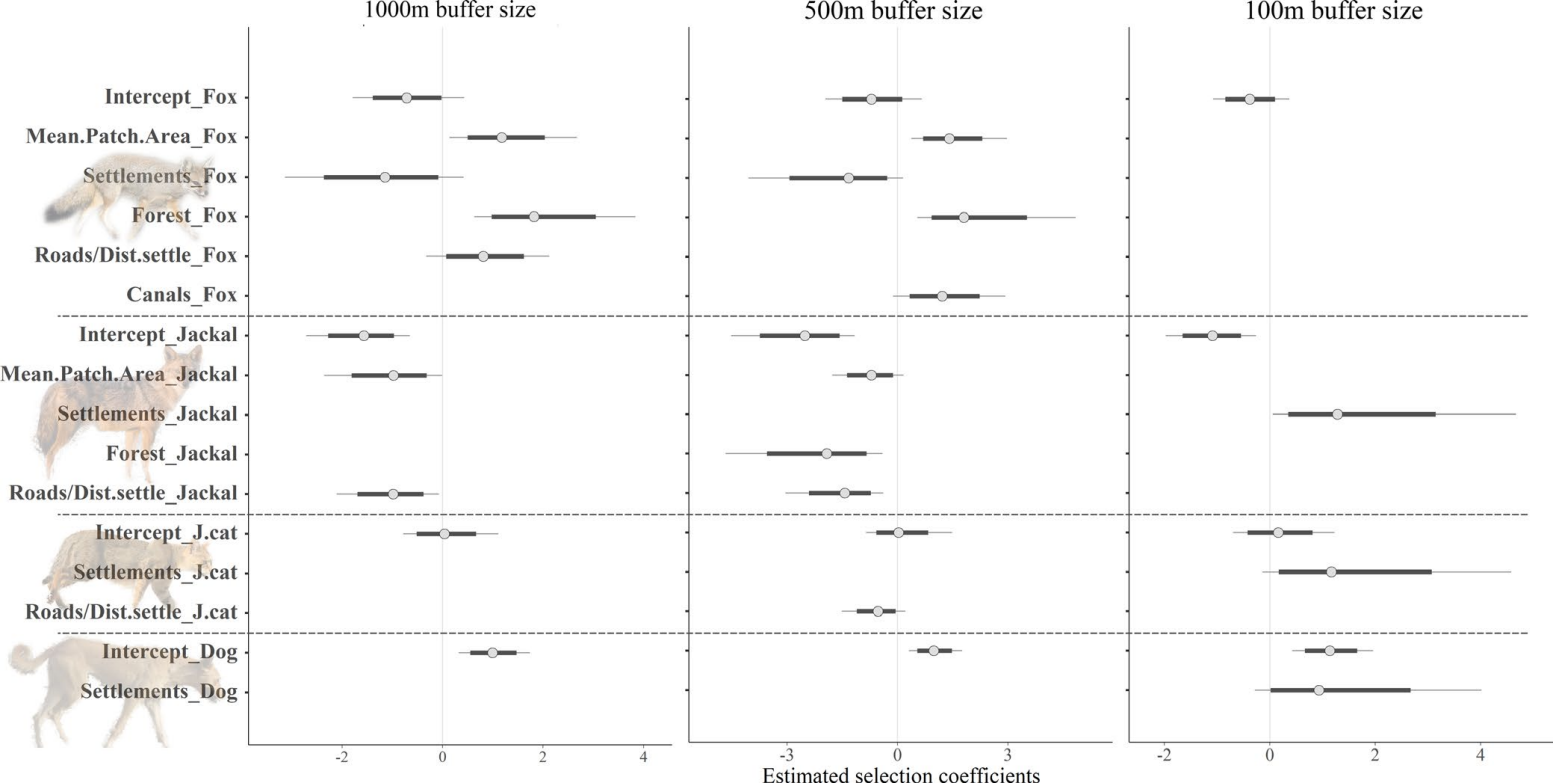
Estimated selection coefficients for the habitat use of Indian fox, jackals, jungle cat, and dogs in a human‐dominated land in Maharashtra, India. Thin lines represent the 95% credible interval, thick lines are the 80% credible interval. Dashed lines separate the different species. Roads/Dist.settle represents the effect of “distance of paved roads” for the 1,000 and the 500 buffer size models and “distance to settlement” for the 100 m buffer size model

Regarding the model coefficients, Indian foxes were positively associated with the mean size of habitat patches on the 500 and the 1,000 m buffer sizes (Figure 2, Table S4). Likewise, occurrence probability of Indian fox increased with the percentage of forestry plantations and the length of paved roads at the 1,000 m buffer size, and with the length of canals at the 500 m buffer size. In contrast, the association between the proportion of human settlements and the probability of Indian fox occurrence at the 500 and 1,000 m buffer sizes was negative (Figure 2, Tables S4). We did not find strong associations at the 100 m buffer size. Occurrence probability of jackals was negatively associated with the mean size of the habitat patch and distance of roads at the 500 and 1,000 m buffer sizes. Additionally, the probability of jackal occurrence was negatively associated with the proportion of forestry patches. Fewer variables had a clear directional association with the occurrence of jungle cats and dogs. However, the occurrence probability of jungle cats was negatively correlated to the length of paved roads at the 500 m scale. Interestingly, jackals, dogs, and jungle cats were positively associated with the proportion of human settlements at 100 m buffer size (Figure 2, Tables S4).

**Figure 3:**
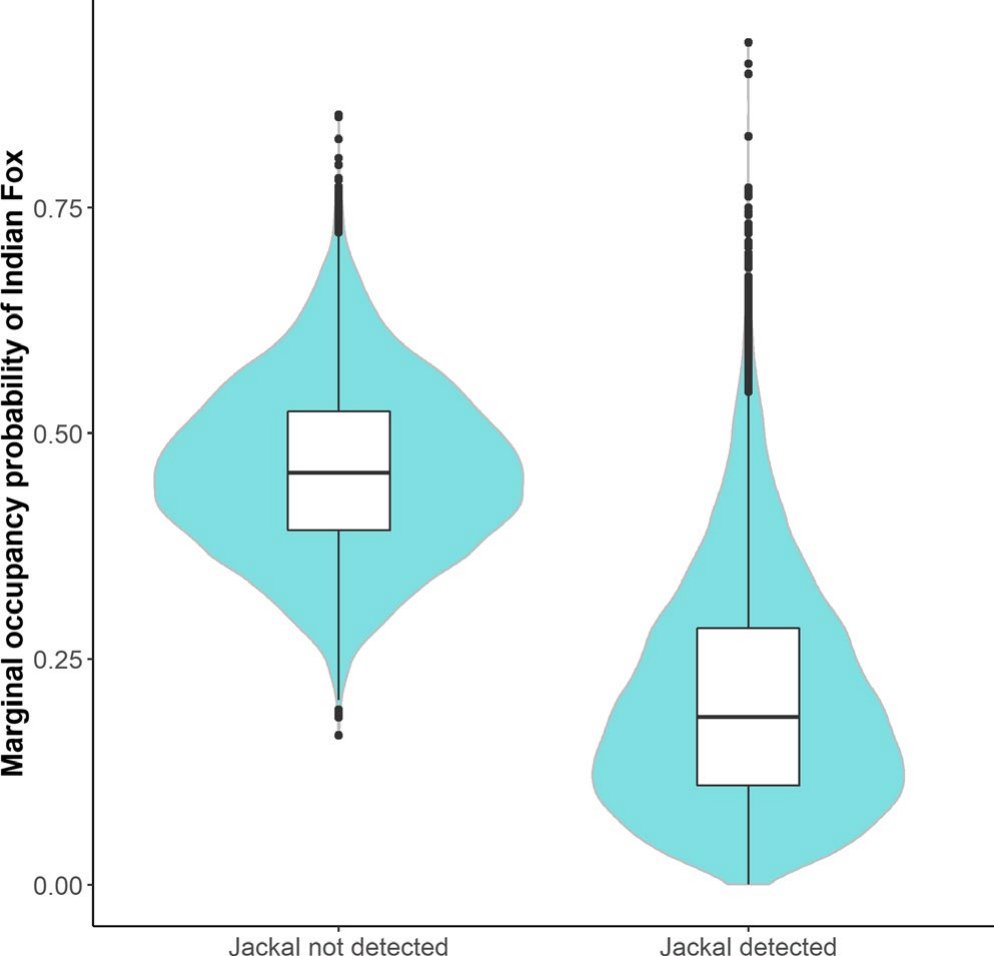
Posterior distributions of the marginal occurrence probability of Indian fox conditional on the detection or non‐detection of Jackal

**Figure 4:**
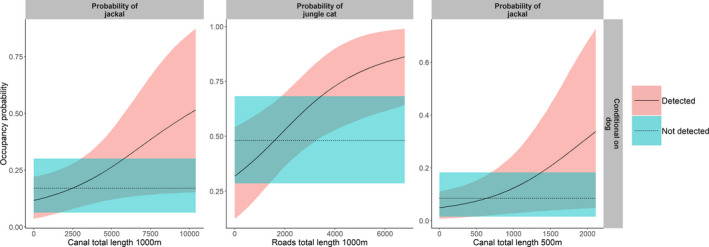
Occurrence probability of jackal conditional on the detection and non‐detection of dogs in relation to the total length of canals at the 500 and 1,000 m buffer size (left and right plots respectively) and the occurrence probability of jungle cat conditional on the detection and non‐detection of dogs in relation to the total length of roads at the 1,000 m buffer size (middle plot). Lines represent the general trend and shaded areas are the 80% credible intervals

Regarding the interaction among species, Indian fox and jackal showed a negative association with each other at the smallest buffer size (Figure 3).The presence of dogs mediated the association of jackals to certain types of infrastructure. When dogs were absent, jackals were positively associated with the presence of canals at the 500 and 1,000 m buffer sizes. However, in the presence of dogs, there was no linear association, and the use of this infrastructure was low (Figure 4). Likewise, in the absence of dogs the accumulated length of roads positively influenced the occurrence of jungle cats (Figure 4).

### 3.2 Activity patterns

**Figure 5:**
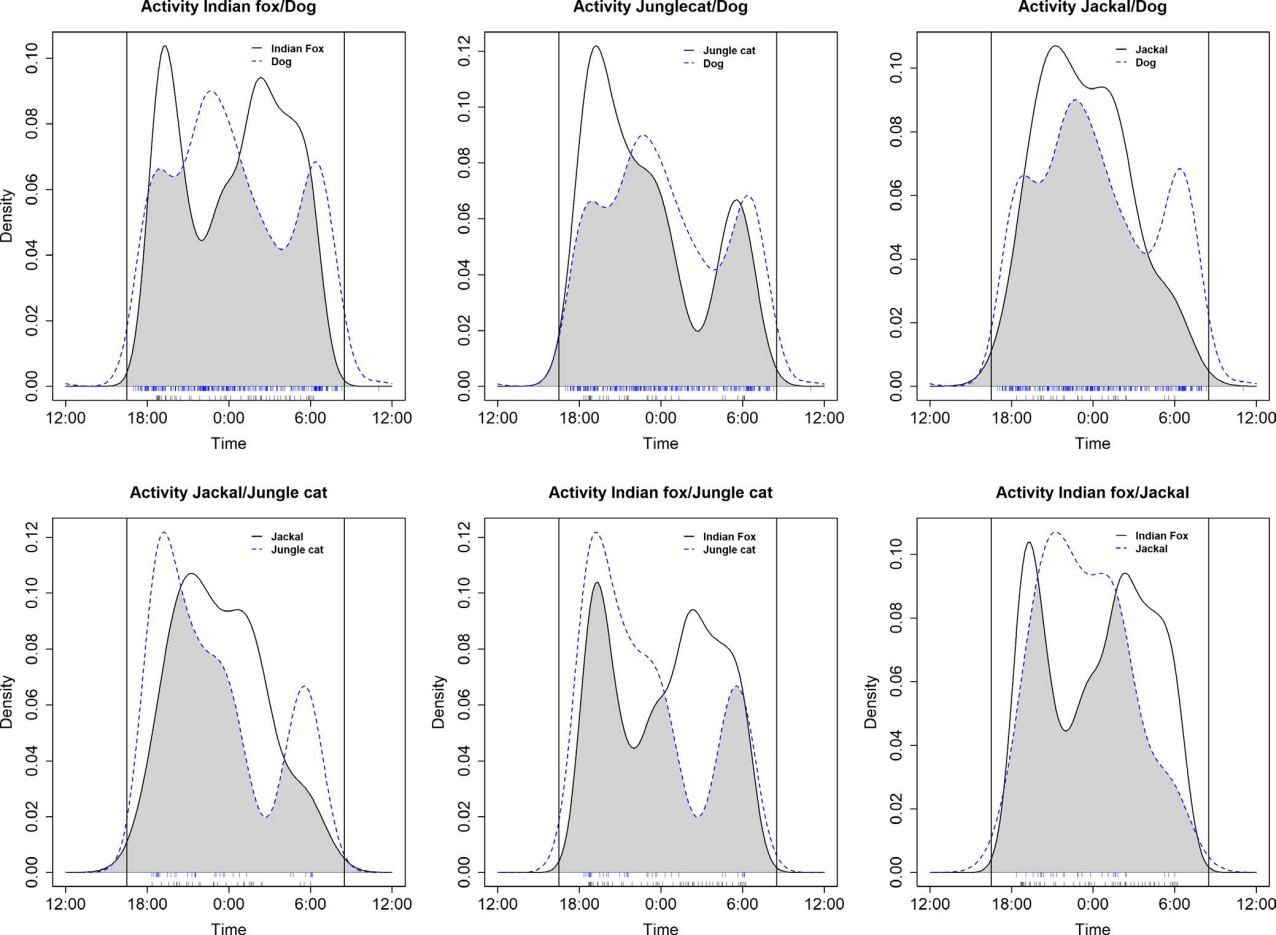
Combined kernel density plots of the nocturnal activity of the different pairs of the study species (i.e., Indian fox, jungle cat, jackal, and domestic dog). Ticks along the *y* axis represent the actual observations for each of the species. Vertical lines comprise the hours when the activity of the animals was monitored (i.e., from dusk until dawn)

The kernel density plots of nocturnal activity showed bimodal distributions for dogs, jungle cats, and Indian fox, and a unimodal distribution for jackals (Figure 5). The coefficients of overlap revealed marked similarities in the activity patterns of all the study species. The largest overlap was between jungle cat and dog (∆ = 0.815, 80% CRI [0.743, 0.883]), followed by the overlap of jackal and dog (∆ = 0.809, 80% CRI [0.737, 0.879]). The overlap between Indian fox and jungle cat was ∆ = 0.769, 80% CRI [0.673, 0.860] and that of the Indian fox and jackal was ∆ = 0.763, 80% CRI [0.668, 0.855]. The smallest overlaps were those between Indian fox and dogs (∆ = 0.759, 80% CRI [0.689, 0.828]) and jackal and jungle cat (∆ = 0.757, 80% CRI [0.669, 0.834]). However, all the credible intervals overlapped with each other, and therefore, differences are statistically weak. Although the overlap coefficients were large, the peaks of activity of Indian fox seemed to coincide with low activity of dogs. Likewise, the peak of nocturnal activity of jackals fell in between the peaks of Indian fox activity (Figure 5). The density plots showed less clear patterns for the other pairs of species (Figure 5).

## 4 DISCUSSION

In this study, we investigated the potential impact of humans (i.e., human infrastructures and domestic dogs) on the co‐occurrence and habitat use of mesocarnivores in a highly dominated landscape in Maharashtra, India. Our results show how, in some carnivore assemblages, smaller species alter their habitat use both spatially and temporally in order to avoid larger competitors. Further, we show that the presence of a species associated with humans, the domestic dog, mediates the influence of human infrastructures on the habitat use of wild carnivores.

In our system, Indian foxes adopted different strategies in order to avoid larger competitors. First, our results showed spatial niche partitioning between Indian foxes and jackals, probably as a result of interference competition. This form of competition is common between carnivore species with marked differences in body size (Palomares & Caro, 1999). For example, red foxes avoided jackals in Israel as a result of a competitive exclusion process (Sheinin, Yom‐Tov, Motro & Geffen, 2006). Typically, this reaction may be stronger when the involved species partially share resources, as might be the case in our study area (Vanak, Thaker & Gompper, 2009). In contrast, the other species in our study used similar habitats. Lesmeister et al. (2015) found a similar carnivore assemblage in North America with little evidence of spatial niche partitioning among species except for the gray fox and the coyote.

Additionally, our results suggest that Indian fox avoided dogs temporally. Temporal niche partitioning occurs, for example, between coyotes and wolves (Atwood & Gese, 2010), or red foxes and dingoes (Mitchell & Banks, 2005). Although we detected no spatial niche separation between the two species, the detectability of foxes decreased as the activity of dogs (number of dog events per trapping night) increased. Moreover, the activity curves showed that Indian fox activity peaked when dog activity was lowest. Vanak and Gompper (2010) suggested that Indian foxes had limited access to human‐derived food and agricultural land due to interference competition with dogs. Moreover, Vanak et al. (2009) found that Indian foxes reduced visitation rates at food trays when exposed to dog odor. Spatial avoidance of dogs may not be possible in our study area since dogs were detected in most of the camera locations (72.5 % of the cameras detected dogs); therefore, Indian fox avoided domestic dogs at a smaller scale by utilizing different activity periods during the night.

Dogs also seemed to affect the use of linear features by jackals and jungle cats. We found that dogs negatively influenced the use of water canals by jackals at the largest scales. Likewise, dogs negatively influenced the use of paved roads by jungle cats. When dogs were absent, Jackals and jungle cats showed a functional response in habitat use in relation to canals and roads respectively (i.e., higher use with higher availability). A similar pattern in the use of roads has been shown at the home range scale, for example, by wolves in Scandinavia (Zimmermann, Nelson, Wabakken, Sand & Liberg, 2014). When the probabilities of interacting with dogs are low, jackals and jungle cats might occasionally use linear infrastructures as means of travel or as a potential source of food (e.g., road kills). However, they seemed to avoid them when the probability of encountering dogs was high. Because of the close association with humans, dogs may extend the effects of human impact in the carnivore communities, especially in non‐protected areas, where carnivores might be more vulnerable (Vanak & Gompper, 2010).

Furthermore, dogs, jackals, and jungle cats showed a positive association with human settlements at the smallest buffer size. A dependency on human food resources probably explains this pattern. Jungle cats are often found in the proximity of villages (Nowell & Jackson, 1996), and jackals are commonly associated with human settlements, where they feed on garbage and other anthropogenic food (Jaeger et al., 2007; Jhala & Moehlman, 2004). Interestingly, this variable was not an important factor explaining the co‐occurrence of these species according to our best model. However, the models at the smallest buffer size that included settlements, showed a weak positive association between this variable and the co‐occurrence of these species, but the credible intervals included zero (logistic coefficients for settlements influencing the co‐occurrence of Jackal/Jungle cat: 1.417, 80% CRI [−0.231, 2.482]; Jackal/Dogs: 0.1488, 80% CRI [−0.153, 2.562]; and Jungle cat/Dogs: 1.512, 80% CRI [−0.174, 2.620]).

The wide credible intervals in the estimates presented in this study might be a result of a small sample size. On the basis of our results and of the results from preliminary studies in the area (supporting that six trapping nights guarantee capture given presence), we believe that the mean estimated coefficients would not have changed with a larger sample size. However, a larger sample size would have narrowed the credible intervals resulting in more robust results. We could not increase the number of trapping night due to logistic constraints, but we recommend a larger number of trapping occasions in future occupancy studies.

The habitat and infrastructure variables associated with each species suggest a gradient of specialization in habitat use. Indian fox showed the most specialized habitat use, followed by the jackal. Jungle cat and dog appeared to be the most opportunistic of the four studied species. Moreover, each species’ preferences depended on the scale of measurement. The differences in the habitat use of a species at different scales are an important aspect of its biology (Mayor et al., 2009). In our study community, it is remarkable that those species with some degree of specialization in their habitat use seemed to be more selective at the largest scales (500 and 1,000 m buffer sizes). Indian foxes selecting their habitat at a landscape scale rather than at a patch scale have been also shown in its denning habits (Punjabi, Chellam & Vanak, 2013). On the contrary, less specialized species seem to be more selective at microhabitat scales (e.g., the association with human settlements at the 100 m buffer size of jackals, jungle cats, and dogs).

In addition, Indian foxes were negatively associated with human settlements and strongly associated with forestry plantations at the largest buffer sizes (500 and 1,000 m). This species is commonly associated with forestry patches, but also to grassland savannah (Vanak & Gompper, 2010). In our study, only two locations had some grassland habitat cover within the buffer areas, and therefore, we included grassland in the fallow land category that consisted mainly of open areas between agricultural fields. Interestingly, the mean area of the habitat patch, representing the homogeneity of the area, was an important predictor of the habitat use of the Indian fox. Further expansion of urban and agricultural areas might increase fragmentation and habitat loss by reducing the size of the natural patches required in their habitat.

Unlike Indian foxes, jackals favored small habitat patches, suggesting a selection for fragmented landscapes where they have access to a variety of food sources. The negative association of jackals with paved roads may reflect a strategy to avoid human interference with a consequent reduction of mortality by road kills. Although we did not detect any effect of agriculture on its habitat use, previous studies have found that jackals use agriculture land, especially sugar cane plantations, as cover during daytime (Jaeger et al., 2007; Poche et al., 1987). The lack of association in our study is probably because this habitat category included both irrigated (e.g., sugarcane) and non‐irrigated cultivation (e.g., maze and grain). A more detailed classification of agricultural land may reveal a stronger association of these two canid species with different agriculture types.

Jungle cats and domestic dogs were detected in a variety of habitat types. They appear to be the most opportunistic of the four studied species and less affected by human activity in this area. This is not surprising, since dogs are closely associated with humans and jungle cats are not considered habitat specialists (Mukherjee et al., 2010), although they show some preferences for open habitats (Mukherjee et al., 2010; Nowell & Jackson, 1996). Jungle cats are also well adapted to cultivated land, and, like the jackal, they use irrigated cultivation like sugarcane as cover (Nowell & Jackson, 1996).

In conclusion, the interactions and habitat use of the species in a mesocarnivore community can be altered by humans through the presence of human infrastructures or by the presence of species commonly associated with humans like free‐ranging domestic dogs. Whereas human influence can benefit some species by food resource subsidization, it may negatively affect others by loss and fragmentation of habitats and by interference interactions with domestic dogs. Therefore, we highlight the importance of acknowledging the potential impact of urbanization not only on single species, but also on the interactions within the community. We recommend focusing future management actions on the community as a whole taking into account interspecific interactions rather than focusing on individual species.

## ACKNOWLEDGMENTS

We thank the Maharashtra Forest Department for support for carrying out this project. We thank A. Lele and P. Panwalkar for assistance in fieldwork. This research was supported in part by the Wellcome Trust/DBT India Alliance Fellowship (Grant number: IA/CPHI/15/1/502028) to ATV, in part by the National Research Foundation of South Africa (Grant Numbers 103659) to ATV, and the Forskning og utvikling funding from Inland Norway University (Project number 46921) to MO.

## Data Availability

Data available from the Dryad Digital Repository: https://doi.org/10.5061/dryad.20j3v31 (Carricondo‐Sanchez, Odden, Kulkarni & Vanak, 2019).
