# Supplementary material for: Scale‐dependent strategies for coexistence of mesocarnivores in human‐dominated landscapes
Source: Biotropica. 2019 Sep 22;51(5):781–91. doi: 10.1111/btp.12705 (PMC8653886; doi:10.1111/btp.12705)

**SUPPORTING INFORMATION**

**TABLES**

**Table 1.** Structure and WAIC values of the occupancy models fitted in the model selection process to explain the habitat use and co-occurrence of mesocarnivores in Maharashtra, India at 1000 m buffer size. Only human infrastructure variables were used to model the interactions among species and therefore, the landscape variables appear shaded.

| **1000 m buffer size** | | | | | | | | | |
| --- | --- | --- | --- | --- | --- | --- | --- | --- | --- |
|  |  | Patch area | Agriculture | Forestry plantation | Fallow land | Human settlements | Distance of road | Distance of canal | Waic |
| **Habitat use** | | | | | | | | | |
|  | *Null* |  |  |  |  |  |  |  |  |
| Model 0 |  |  |  |  |  |  |  |  | 727.265 |
|  | *Indian fox* |  |  |  |  |  |  |  |  |
| Model 1 |  | * | * | * | * | * | * | * | 716.020 |
| Model 2 |  | * |  | * |  | * | * | * | 713.767 |
| Model 3 |  | * |  | * |  | * | * |  | 712.644 |
|  | *Jackal* |  |  |  |  |  |  |  |  |
| Model 4 |  | * | * | * | * | * | * | * | 723.365 |
| Model 5 |  |  |  | * |  |  | * |  | 716.368 |
|  | *Jungle cat* |  |  |  |  |  |  |  |  |
| Model 6 |  | * | * | * | * | * | * | * | 731.259 |
|  | *Dog* |  |  |  |  |  |  |  |  |
| Model 7 |  | * | * | * | * | * | * | * | 734.825 |
|  | Multi-species |  |  |  |  |  |  |  |  |
| Model 8 | *Indian fox* | * |  | * |  | * | * |  | 708.345 |
|  | *Jackal* |  |  | * |  |  | * |  |  |
|  | *Jungle cat* |  |  |  |  |  |  |  |  |
|  | *Dog* |  |  |  |  |  |  |  |  |
| **Interactions (built upon multi-species model)** | | | | | | | | | |
| *Null interactions* | |  |  |  |  |  |  |  |  |
| Model 9 |  |  |  |  |  |  |  |  | 715.341 |
|  | *Fox - Jackal* |  |  |  |  |  |  |  |  |
| Model 10 |  |  |  |  |  | * | * | * | 716.317 |
|  | *Fox – Jun cat* |  |  |  |  |  |  |  |  |
| Model 11 |  |  |  |  |  | * | * | * | 719.517 |
|  | *Fox - Dog* |  |  |  |  |  |  |  |  |
| Model 12 |  |  |  |  |  | * | * | * | 719.293 |
| *Jackal – Jun cat* | |  |  |  |  |  |  |  |  |
| Model 13 |  |  |  |  |  | * | * | * | 720.441 |
|  | *Jackal - Dog* |  |  |  |  |  |  |  |  |
| Model 14 |  |  |  |  |  | * | * | * | 719.781 |
| Model 15 |  |  |  |  |  |  |  | * | 716.466 |
|  | *Jun cat - Dog* |  |  |  |  |  |  |  |  |
| Model 16 |  |  |  |  |  | * | * | * | 718.442 |
| Model 17 |  |  |  |  |  |  | * |  | 714.303 |
| **Combined final model** | | | | | | | | | |
| Model 18 | *Indian fox* | * |  | * |  |  | * |  |  |
|  | *Jackal* |  |  | * |  | * | * |  |  |
|  | *Jungle cat* |  |  |  |  |  |  |  |  |
|  | *Dog* |  |  |  |  |  |  |  | 710.553 |
|  | *Jackal - Dog* |  |  |  |  |  |  | * |  |
|  | *Jun cat - Dog* |  |  |  |  |  | * |  |  |

**Table 2.** Structure and WAIC values of the occupancy models fitted in the model selection process to explain the habitat use and co-occurrence of mesocarnivores in Maharashtra, India at 500 m buffer size. Only human infrastructure variables were used to model the interactions among species and therefore, the landscape variables appear shaded.

| **500 m buffer size** | | | | | | | | | | |
| --- | --- | --- | --- | --- | --- | --- | --- | --- | --- | --- |
|  |  | Patch area | | Agriculture | Forestry plantation | Fallow land | Human settlements | Distance of road | Distance of canal | Waic |
| **Habitat use** | | | | | | | | | | |
|  | *Null* | |  |  |  |  |  |  |  |  |
| Model 0 |  | |  |  |  |  |  |  |  | 727.265 |
|  | *Indian fox* | |  |  |  |  |  |  |  |  |
| Model 1 |  | | * | * | * | * | * | * | * | 703.154 |
| Model 2 |  | | * |  | * |  | * |  | * | 703.174 |
| Model 3 |  | |  |  |  |  |  |  |  |  |
|  | *Jackal* | |  |  |  |  |  |  |  |  |
| Model 4 |  | | * | * | * | * | * | * | * | 711.921 |
| Model 5 |  | | * |  | * |  |  | * |  | 707.450 |
|  | *Jungle cat* | |  |  |  |  |  |  |  |  |
| Model 6 |  | | * | * | * | * | * | * | * | 730.554 |
| Model 7 |  | |  |  |  |  |  | * |  | 721.438 |
|  | *Dog* | |  |  |  |  |  |  |  |  |
| Model 8 |  | | * | * | * | * | * | * | * | 731.382 |
|  | Multi-species | |  |  |  |  |  |  |  |  |
| Model 9 | *Indian fox* | | * | * | * | * | * | * | * | 690.783 |
|  | *Jackal* | | * |  | * |  |  | * |  |  |
|  | *Jungle cat* | |  |  |  |  |  | * |  |  |
|  | *Dog* | |  |  |  |  |  |  |  |  |
| **Interactions (built upon multi-species model)** | | | | | | | | | | |
| *Null interactions* | | |  |  |  |  |  |  |  |  |
| Model 10 |  | |  |  |  |  |  |  |  | 699.267 |
|  | *Fox - Jackal* | |  |  |  |  |  |  |  |  |
| Model 11 |  | |  |  |  |  | * | * | * | 700.074 |
|  | *Fox – Jun cat* | |  |  |  |  |  |  |  |  |
| Model 12 |  | |  |  |  |  | * | * | * | 703.596 |
|  | *Fox - Dog* | |  |  |  |  |  |  |  |  |
| Model 13 |  | |  |  |  |  | * | * | * | 704.694 |
| *Jackal – Jun cat* | | |  |  |  |  |  |  |  |  |
| Model 14 |  | |  |  |  |  | * | * | * | 701.877 |
| Model 15 |  | |  |  |  |  |  |  | * | 699.416 |
|  | *Jackal - Dog* | |  |  |  |  |  |  |  |  |
| Model 16 |  | |  |  |  |  | * | * | * | 700.249 |
| Model 17 |  | |  |  |  |  |  |  | * | 698.190 |
|  | *Jun cat - Dog* | |  |  |  |  |  |  |  |  |
| Model 17 |  | |  |  |  |  | * | * | * | 704.036 |
| **Combined final model** | | | | | | | | | | |
| Model 18 | *Indian fox* | | * | * | * | * | * | * | * |  |
|  | *Jackal* | | * |  | * |  |  | * |  |  |
|  | *Jungle cat* | |  |  |  |  |  | * |  | 691.399 |
|  | *Dog* | |  |  |  |  |  |  |  |  |
|  | *Jackal - Dog* | |  |  |  |  |  |  | * |  |

**Table 3.** Structure and WAIC values of the occupancy models fitted in the model selection process to explain the habitat use and co-occurrence of mesocarnivores in Maharashtra, India at 100 m buffer size. Only human infrastructure variables were used to model the interactions among species and therefore, the landscape variables appear shaded.

| **100 m buffer size** | | | | | | | | | |
| --- | --- | --- | --- | --- | --- | --- | --- | --- | --- |
|  |  | Patch area | Agriculture | Forestry plantation | Fallow land | Human settlements | Distance to settlements | Distance of canal | Waic |
| **Habitat use** | | | | | | | | | |
|  | *Null* |  |  |  |  |  |  |  |  |
| Model 0 |  |  |  |  |  |  |  |  | 727.265 |
|  | *Indian fox* |  |  |  |  |  |  |  |  |
| Model 1 |  | * | * | * | * | * | * | * | 724.915 |
|  | *Jackal* |  |  |  |  |  |  |  |  |
| Model 2 |  | * | * | * | * | * | * | * | 717.296 |
| Model 3 |  |  |  |  |  | * |  |  | 719.817 |
|  | *Jungle cat* |  |  |  |  |  |  |  |  |
| Model 4 |  | * | * | * | * | * | * | * | 727.517 |
| Model 6 |  |  |  |  |  | * |  |  | 719.964 |
|  | *Dog* |  |  |  |  |  |  |  |  |
| Model 7 |  | * | * | * | * | * | * | * | 726.800 |
| Model 8 |  |  |  |  |  | * |  |  | 720.597 |
| Multi-species | |  |  |  |  |  |  |  |  |
| Model 9 | *Indian fox* |  |  |  |  |  |  |  |  |
|  | *Jackal* |  |  |  |  | * |  |  | 717.080 |
|  | *Jungle cat* |  |  |  |  | * |  |  |  |
|  | *Dog* |  |  |  |  | * |  |  |  |
| **Interactions (built upon multi-species model)** | | | | | | | | | |
| *Null interactions* | |  |  |  |  |  |  |  |  |
| Model 10 |  |  |  |  |  |  |  |  | 723.417 |
|  | *Fox - Jackal* |  |  |  |  |  |  |  |  |
| Model 11 |  |  |  |  |  | * | * | * | 725.282 |
|  | *Fox – Jun cat* |  |  |  |  |  |  |  |  |
| Model 12 |  |  |  |  |  | * | * | * | 725.027 |
|  | *Fox - Dog* |  |  |  |  |  |  |  |  |
| Model 13 |  |  |  |  |  | * | * | * | 730.010 |
| *Jackal – Jun cat* | |  |  |  |  |  |  |  |  |
| Model 14 |  |  |  |  |  | * | * | * | 725.200 |
|  | *Jackal - Dog* |  |  |  |  |  |  |  |  |
| Model 15 |  |  |  |  |  | * | * | * | 724.671 |
|  | *Jun cat - Dog* |  |  |  |  |  |  |  |  |
| Model 16 |  |  |  |  |  | * | ***** | * | 728.228 |
| **Combined final model** | | | | | | | | | |
| Model 17 | *Indian fox* |  |  |  |  |  |  |  |  |
|  | *Jackal* |  |  |  |  | * |  |  | 716.247 |
|  | *Jungle cat* |  |  |  |  | * |  |  |  |
|  | *Dog* |  |  |  |  | * |  |  |  |
|  | *Fox - Jackal* |  |  |  |  |  |  |  |  |

**Table 4.** Model coefficients of the best occupancy models for each buffer size explaining the habitat use and co-occurrence of mesocarnivores in Maharashtra, India. 10% and 90% credible intervals are presented. Rhat is the Gelman & Rubin’s convergence diagnosis, and Delta WAIC represents the deviation in WAIC values from the null model.

|  | **Estimate** | **10% LowerCRI** | **90% UpperCRI** | **Rhat** | **WAIC** | **Delta WAIC** |
| --- | --- | --- | --- | --- | --- | --- |
| **Model 1000 m** |  |  |  |  | 708.345 | 18.920 |
| *Fox* |  |  |  |  |  |  |
| Intercept | -0.706 | -1.389 | -0.023 | 0.999 |  |  |
| Mean patch area | 1.235 | 0.497 | 2.037 | 0.999 |  |  |
| Settlements | -1.2 | -2.362 | -0.085 | 1 |  |  |
| Forestry | 1.939 | 0.977 | 3.049 | 0.999 |  |  |
| Roads | 0.835 | 0.072 | 1.619 | 0.999 |  |  |
| *Jackal* |  |  |  |  |  |  |
| Intercept | -1.601 | -2.278 | -0.968 | 1 |  |  |
| Forestry | -1.031 | -1.809 | -0.317 | 1 |  |  |
| Roads | -1.017 | -1.695 | -0.378 | 1 |  |  |
| *Jungle cat* | 0 | 0 | 0 |  |  |  |
| Intercept | 0.069 | -0.518 | 0.669 | 0.999 |  |  |
| *Dog* |  |  |  |  |  |  |
| Intercept | 1.001 | 0.549 | 1.473 | 0.999 |  |  |
| **w/ Interactions** |  |  |  |  | 710.553 | 16.712 |
| *Jackal: Dog* |  |  |  |  |  |  |
| Intercept | 0.061 | -0.909 | 1.059 | 0.999 |  |  |
| Canals | 0.567 | 0.014 | 1.15 | 1 |  |  |
| *Jungle Cat : Dog* |  |  |  |  |  |  |
| Intercept | 0.112 | -0.842 | 1.056 | 0.999 |  |  |
| Roads | 0.745 | 0.174 | 1.343 | 0.999 |  |  |
| **Model 500 m** |  |  |  |  | 690.783 | 36.482 |
| *Fox* |  |  |  |  |  |  |
| Intercept | -0.694 | -1.499 | 0.135 | 1 |  |  |
| Mean patch area | 1.473 | 0.702 | 2.307 | 0.999 |  |  |
| Settlements | -1.491 | -2.931 | -0.276 | 1 |  |  |
| Forestry | 2.045 | 0.931 | 3.521 | 1 |  |  |
| Canal | 1.26 | 0.329 | 2.238 | 0.999 |  |  |
| *Jackal* |  |  |  |  |  |  |
| Intercept | -2.602 | -3.739 | -1.571 | 1.001 |  |  |
| Mean patch area | -0.732 | -1.371 | -0.117 | 1.000096 |  |  |
| Forestry | -2.083 | -3.545 | -0.835 | 1.001 |  |  |
| Roads | -1.511 | -2.402 | -0.722 | 1 |  |  |
| *Jungle cat* |  |  |  |  |  |  |
| Intercept | 0.1 | -0.571 | 0.832 | 1 |  |  |
| Roads | -0.556 | -1.1 | -0.046 | 1 |  |  |
| *Dog* |  |  |  |  |  |  |
| Intercept | 1.002 | 0.543 | 1.482 | 0.999 |  |  |
| **w/ Interactions** |  |  |  |  | 691.399 | 35.866 |
| *Jackal: Dog* |  |  |  |  |  |  |
| Intercept | -0.085 | -1.033 | 0.88 | 0.999 |  |  |
| Canals | 0.747 | 0.047 | 1.472 | 0.999 |  |  |
| **Model 100 m** |  |  |  |  | 717.080 | 10.185 |
| *Fox* |  |  |  |  |  |  |
| Intercept | -0.376 | -0.845 | 0.101 | 0.999 |  |  |
| *Jackal* |  |  |  |  |  |  |
| Intercept | -1.093 | -1.654 | -0.545 | 0.999 |  |  |
| Settlements | 1.567 | 0.351 | 3.145 | 1 |  |  |
| *Jungle cat* |  |  |  |  |  |  |
| Intercept | 0.187 | -0.421 | 0.813 | 1 |  |  |
| Settlements | 1.433 | 0.169 | 3.07 | 1 |  |  |
| *Dog* |  |  |  |  |  |  |
| Intercept | 1.153 | 0.666 | 1.659 | 1 |  |  |
| Settlements | 1.179 | 0.015 | 2.668 | 0.999 |  |  |
| **w/ Interactions** |  |  |  |  | 716.247 | 11.018 |
| *Indian Fox: Jackal* |  |  |  |  |  |  |
| Intercept | -1.375 | -2.592 | -0.225 | 0.999 |  |  |

**FIGURES**

**Figure 1.** (A) Boxplots depicting the detection probability of Indian foxes, jackals, jungle cats and dogs when the camera locations are placed either on or outside a trail or natural path. (B) Effect of the number of dogs each trapping night on the detection probability of foxes, jackals and jungle cats where the solid line represent the general trend and the shaded area the 80% credible interval.


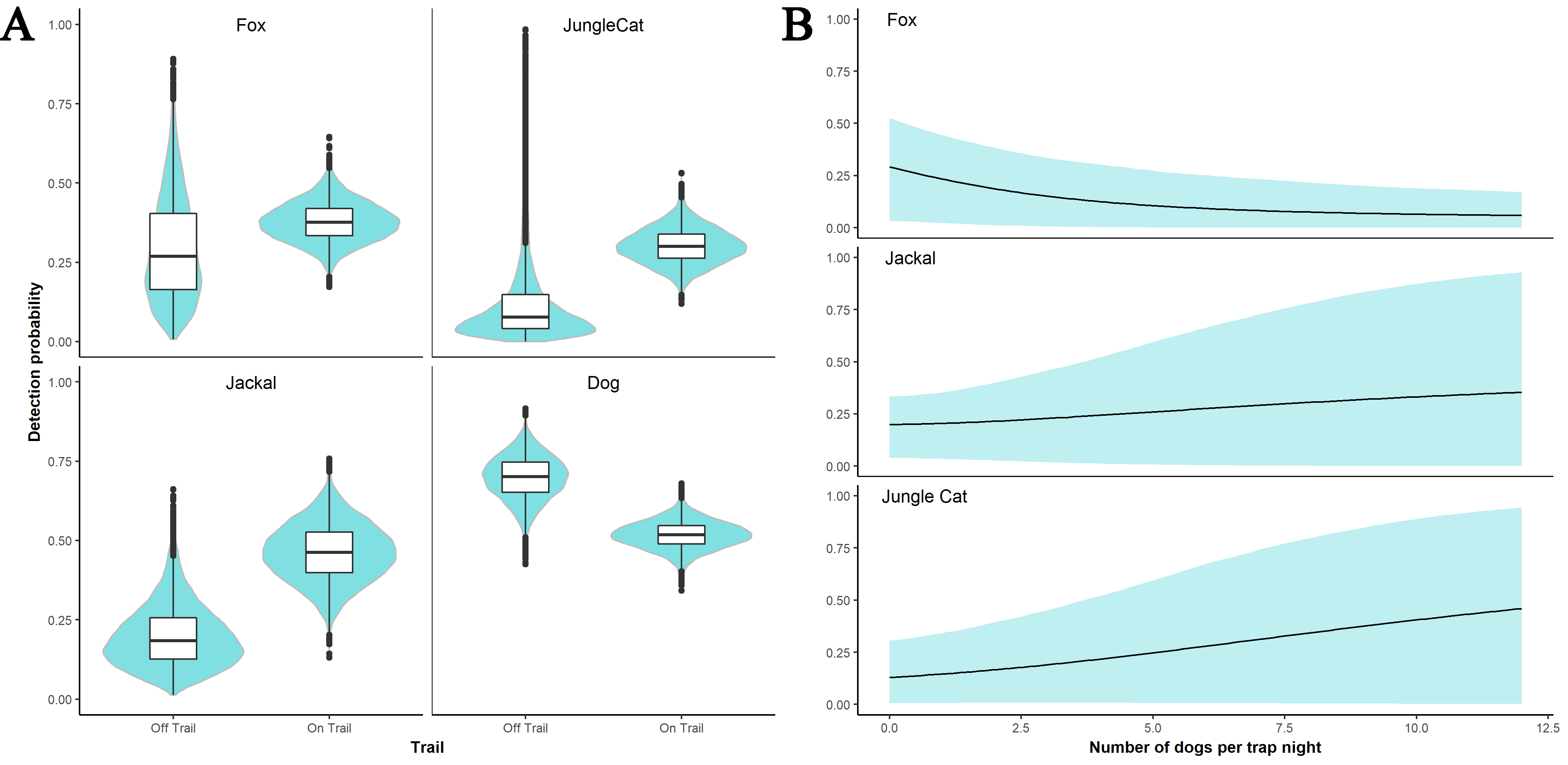

Supplement: Supplementary file 1 [file BTP-51-781-s001.docx]
